# Strategies to Promote Empowerment Status of Breast Cancer Women

**DOI:** 10.1155/2024/3305399

**Published:** 2024-02-05

**Authors:** Mohammad Amin Bahrami, Shahrzad Tabari, Sedigheh Tahmasebi, Vahid Zangouri, Ramin Ravangard

**Affiliations:** ^1^Health Human Resources Research Center, Department of Health Services Management, School of Management and Medical Information Sciences, Shiraz University of Medical Sciences, Shiraz, Iran; ^2^Student Research Committee, Department of Health Services Management, School of Management and Medical Information Sciences, Shiraz University of Medical Sciences, Shiraz, Iran; ^3^Breast Diseases Research Center, Shiraz University of Medical Sciences, Shiraz, Iran

## Abstract

**Background:**

As the second leading cause of death in women in the world, breast cancer has several physical and psychological effects. Nowadays, nonclinical approaches such as patient empowerment have been considered by physicians along with clinical care. Given the increasing number of breast cancer women worldwide, promoting the empowerment of these patients is one of the key factors affecting their survival and quality of life. Therefore, because of no comprehensive research on the empowerment needs and related improvement strategies, this study is aimed at determining the empowerment status of breast cancer patients referred to the Shahid Motahari Breast Cancer Clinic in Iran, Shiraz, and at providing strategies to improve their empowerment in 2021.

**Methods:**

This applied study was conducted in two phases. In the quantitative phase, 310 Cancer-Related Patient Empowerment Scale questionnaires (Persian format) were distributed among the studied patients selected through the random sampling method in the clinic, and the items with “unacceptable status” became the basis for determining the empowerment strategies through the scoping review and semistructured interviews with 22 medical staff and patients through the thematic analysis. The collected data were analyzed using the SPSS 20.0 and MAXQDA10 software.

**Results:**

The mean score of the participants' empowerment strategies was 3.58. The results showed that trust in the physician, family support, and spiritual beliefs could affect the empowerment of the studied patients. Moreover, the participants needed empowerment strategies in 11 scale items with unacceptable status, for which 46 strategies were determined in the scoping review and interview phase.

**Conclusion:**

The results of this study provided useful strategies for empowering breast cancer patients, the most important of which were classified into five categories of financial support, informational support, interaction with the physician, occupational support, and complementary therapies, the use of which by the stakeholders could help to improve the patients' quality of life while improving their empowerment.

## 1. Introduction

Cancer is one of the leading causes of disorders, mortality, and disability worldwide [1] and has experienced an alarming growth in the last two decades and is considered as an urgent health problem of modern life. Its negative effects invariably affect the physical, psychological, social, and economic aspects of human life and continue to be of great concern to experts [2]. Among all cancers, breast cancer is the most common type of malignancy among women in developed countries. It is the most common type of cancer among women aged 40 to 44 and the first cause of cancer-related death in women, which is responsible for 19% of cancer-related deaths [3]. Today, one in nine women in developed countries has experienced breast cancer during her life, but in less developed countries, one in 20 women has had such an experience [4]. According to the latest statistics of the Cancer Research Center in Iran, about 8500 new cases of breast cancer are recorded annually in the country and 1400 women die from the disease [5].

When receiving the diagnosis and tolerating breast cancer treatment, women experience a great deal of vulnerability through feelings of insecurity, anxiety, depression, anger, fear of treatment, femininity weakness, worry about survival and health, and loss of control [6], which cause severe psychological effects, and they need to be supported [7]. Breast cancer affects the women's appearance, distorts their self-image, and influences their personal-family and social interactions; thus, it is necessary to help these patients by empowering them to adapt and meet their impaired needs [8].

Considering the increase in the number of women suffering from breast cancer all over the world, improving the empowerment of these patients is one of the important factors affecting their survival. Also, due to the important role of women in development, empowering women with breast cancer, as a relatively large group, is necessary for the sustainable development of a country [9].

Empowerment is the main practical solution to improving the health level of patients and their families. Empowerment is defined as involving patients and their families in making decisions about their health and well-being [10]. Patient empowerment is a process in which patients increase and strengthen their required resources and gain the ability to control their lives, meet their needs, and solve their problems. An empowered patient is an informed one who feels as responsible for his/her own health as possible. Empowerment also leads to access to appropriate and quality care, improved quality of life, increased responsiveness, better interactions with medical staff, increased satisfaction, better response to therapy, prevention of complications, and a positive attitude toward the disease [9]. Therefore, developing and implementing an empowerment program to increase the awareness and knowledge as well as self-efficacy of the patients can lead to behavioral self-control and adoption of preventive behaviors, resulting in improved quality of life [11].

Numerous studies have been conducted on the empowerment of breast cancer patients so far. The results of a study on the factors affecting empowerment showed that the implementation of a family-centered empowerment model was effective in improving the functional dimensions of life quality of breast cancer patients undergoing chemotherapy [12]. In another study, three main categories of empowerment needs from the perspective of the patients included access to information through initial empowerment programs (including timely and comprehensive information, coordination and continuity of information, and easy and full-time access to information), new beliefs on implementation of empowerment programs, and development of new skills for effective implementation of empowerment programs (such as communication skills, expression of needs and feelings, and use of the Internet) [9]. It was stated in another study that breast self-examination was one of the most effective methods for early detection of breast cancer in order to receive timely treatment, and this could be achieved by education and enhancement of women's skills [13]. Furthermore, membership in a support group on Twitter social media increased the perceived knowledge of breast cancer patients and decreased their anxiety [14, 15]. Regarding the effects of empowerment, the results of a study showed that empowerment of breast cancer survivors significantly affected their quality of life, and participation in a self-help group had a remarkable effect on their sense of empowerment, which in turn affected their quality of life [13, 16, 17].

Although empowerment is one of the most important approaches in caring for patients, especially breast cancer patients, no comprehensive research has been conducted to identify the empowerment needs of these patients to offer strategies to improve such needs. Thus, the present study is aimed at determining the empowerment status of breast cancer patients referred to the Shahid Motahari Breast Cancer Clinic in Iran, Shiraz, and at providing strategies to improve their empowerment in 2021.

## 2. Materials and Methods

This was an applied study conducted in two phases, including a quantitative phase and a scoping review and interviews in 2021 in Shahid Motahari Breast Cancer Clinic in Iran, Shiraz. This clinic has been established to examine and treat breast cancer patients and hold educational classes for them, and patients visit it daily.

### 2.1. Phase 1: The Quantitative Phase

In the first phase, the empowerment status of breast cancer women was determined quantitatively by the use of the Cancer-Related Patient Empowerment Scale developed by Bulsara et al. [18]. The scale contained 28 items on 14 dimensions (each of which with two items), including resources, information, participation in decision-making, family support, support of friends, interaction with the physician, patient perception of the physician's ability to manage the disease, patient perception of health professionals' willingness to let him/her participate in decision-making, complementary therapies, spiritual beliefs, acceptance and adaptation to disease, patient perception of usefulness to friends, patient perception of usefulness to family, and having a job [19].

The study population in this phase consisted of the breast cancer patients referred to the studied clinic. Because the number of samples required for this phase had to be between 5 and 15 samples for each questionnaire item [20], 10 samples were selected for each item in the present study. Therefore, considering that the number of items in the questionnaire was 28 and assuming 10% patient dropout, 310 patients willing to participate in the study were selected using the random sampling method based on the patients' record numbers in the clinic.

Given that the Persian version of the questionnaire was not available, it was prepared using the translate-retranslate method of the original questionnaire [21]. The face validity of the questionnaire was confirmed using the opinion of 10 experts, including four oncologists, three general surgeons, and three healthcare management specialists. The content validity of the questionnaire was also confirmed using the content validity ratio (CVR), designed by Lawshe, such that 10 experts mentioned were explained the objectives of the study and were provided operational definitions related to the content of the items. They were then asked to rate the items based on a 3- point Likert scale as “item is necessary,” “item is useful but not necessary,” and “item is not necessary.” All items had CVR > 0.62, and a total CVR score of 0.78 was obtained [22, 23]. To determine the reliability, the questionnaire was completed by 20 women with breast cancer and was confirmed using Cronbach's alpha coefficient (*α* = 0.89). The participants responded to the items of the questionnaire based on a five-point Likert scale, that is, 1 referred to as “very low” and 5 as “very high.” After completing the questionnaires, the mean score of each item and the mean score of the total items of the questionnaire were calculated. Then, the items whose mean score was lower than the mean score of the total items were called “unacceptable status” and became the basis for determining the empowerment needs of the research sample in the second phase.

The data collected in the first phase of the study were analyzed using SPSS 20.0 software as well as the statistical tests including the Pearson correlation coefficient, as well as independent *T*-test and ANOVA tests.

### 2.2. Phase 2: The Scoping Review and Semistructured Interviews

In order to extract the most important strategies for empowering women with breast cancer referred to the studied clinic, the scoping review and semistructured interviews were used to provide strategies for items with a mean of less than 3.58, i.e., the mean score of the total items.

In the scoping review, all related articles available in the databases such as Web of Science, Scopus, PubMed, Embase, ProQuest, Google Scholar, Magiran, IranDoc, and SID, without a time limit until April 31, 2021, were searched. The inclusion criteria were the published articles related to the empowerment strategies for breast cancer women in Persian and English and access to the full texts, and the exclusion criteria included gray literature, letters to the editor, reports, notes, and conference papers.

In the scoping review, the researchers used the Persian version of Preferred Reporting Items for Systematic Reviews and Meta-Analyses (PRISMA) [24, 25]. The data collection tool in this phase was the data extraction form containing the article title, first author, year of publication, place of study, research method, and empowerment strategies for breast cancer women.

The search strategy was confirmed by two other members of the research team through repeated searches. The search results were entered into the EndNote X9 software. After removing duplicates, the titles and abstracts of the articles were screened by two members of the research team (M.A.B and Sh.T.) based on the inclusion and exclusion criteria. The full texts of the articles were studied by one of the researchers (Sh.T.), and the extracted information was confirmed by another member of the research team (R.R.).

The search strategy and search strings used to find the articles have been shown in the supplementary section (Tables S1 and S2).

In the semistructured interview, the study population included 22 participants, including specialists in the field of breast cancer (including gynecologic oncologists, internists practicing oncology, and breast cancer surgeons), nurses experienced in caring for breast cancer patients and breast cancer women, and experts in the fields of health education, health services management, and health policies working in the Shahid Motahari Clinic and Vice Chancellor of Shiraz University of Medical Sciences, who were selected through the purposeful sampling *heterogeneous* method. The main inclusion criteria were as follows: having experience in the treatment of breast cancer patients, having an education degree and experience as well as published scientific materials on breast cancer care, having experience in making decisions or policies on breast cancer care, having been diagnosed with breast cancer, and willingness to participate in the study.

In order to conduct semistructured interviews, a mobile voice recorder and the interview guide prepared by the researchers were used. The questions of the semistructured interviews were written based on some items of the Cancer-Related Patient Empowerment Scale whose status had been identified as “unacceptable” in the first phase. In other words, the items whose mean scores were lower than the mean score of all items were identified as “unacceptable status” and were considered as the basis for determining the empowerment strategies for the samples in the second phase. The deficiencies of the prepared guide were resolved by conducting initial interviews with three participants and using the comments of the research team. The guide contained 13 questions (related to the empowerment of breast cancer women) according to the number of items with “unacceptable status.”

To conduct the interviews, the samples were talked to in person, and if they were willing, the time of the interviews was set as agreed by the parties. Regarding the breast cancer women, the time of the interviews was set through coordination with the head of the Shahid Motahari Clinic. Before the interviews, written informed consent was obtained from the participants. At the appointed time, the researcher went to the participants' places for the interviews and explained the research objectives and the confidentiality of the information before the interviews. The interviews were recorded with the knowledge of the interviewees, and the note-taking method was used during the interviews. The average time allotted for each interview was 15 minutes. After the end of each interview, it was listened to and transcribed word by word in the Microsoft Word 2016 software. The interview process continued until data saturation was reached, and during the analysis, additional interviews were conducted if necessary. In case some individuals were reluctant to continue the interviews at any stage, they were excluded from the study, and all data related to them were removed from the researchers' files. To maintain the confidentiality of the data, the quotes were coded.

The data collected in the scoping review and interviews phase were analyzed using MAXQDA10 software and through the thematic analysis method, a method for analyzing the qualitative data. In the thematic analysis method, the meaning units related to the research objectives are extracted from the texts. Then, they are merged with each other based on the meaning similarity and overlapping with each other and are reported in the form of main themes and subthemes [26]. Thus, the empowerment strategies for breast cancer women were extracted as the main themes from the studies and the transcripts of the interviews, and the results were reported in tables. In order to ensure the trustworthiness of the obtained data, the Lincoln and Guba criteria including credibility, dependability, transferability, and confirmability were used as well [21].

### 2.3. Ethical Considerations

This study was approved by the Shiraz University of Medical Sciences Ethics Committee (Code: IR.SUMS.REC.1399.642). At the stages of completing the questionnaire and doing the interviewees, the personal information of the patients and interviewees was available to only one member of the research group (Sh.T) who was responsible for collecting the data. In the phases of this research, the personal characteristics of the participants and their answers remained confidential, and all recorded reports were presented based on coding. All the participants were aware of the research process and were allowed to withdraw from the study whenever they wished. In addition, honesty and trustworthiness were observed in the selection of articles, reporting the findings, and citations to texts.

## 3. Results

The results of analyzing the empowerment scale in the quantitative phase showed that the mean age of the patients was 48.7 ± 10.26 years, and the mean duration of their disease was 3.63 ± 3.52 years. The mean monthly income of the patients participating in the study was 30.72 ± 1.93 million rials (equal to 731 USD) (Table 1).

Furthermore, most of the patients studied were married (81.9%) and lived with their families (96.5%). They were not heads of households (88.1%), had elementary school education (75.5%), and were unemployed (85.5%). Most of the patients were in stage 2 of the disease (52.3%), had no history of other diseases (78.1%), had no job before the disease (80.1%), and were mostly nonlocal (54.2%) (Table 2).

The results also showed that the highest mean score of the scale items as stated by the patients was that of “I believe my physician is really good” (4.42 ± 0.60) and the lowest was that of “I use complementary therapies (such as traditional medicine or rehabilitation)” (1.88 ± 0.94). In addition, the mean scores of the dimensions of patient empowerment showed that the highest and lowest mean scores belonged to “interaction with the physician” (4.36 ± 0.53) and “complementary therapies” (1.99 ± 0.88) (Table 3).

According to the results, 11 items on the patient empowerment had means less than the mean score of all items in different dimensions and were assigned the term “unacceptable status” (Table 4).

The results also showed that among the studied demographic variables, there were statistically significant differences only between the mean total score of patient empowerment and education level (*P* < 0.001), monthly income (*P* < 0.001), employment status (*P* < 0.001), and having a job before the disease (*P* < 0.001). In other words, the patients with academic education levels, those with higher income, and the patients who were employed before and after the disease diagnosis had higher mean empowerment scores.

**Figure 1:**
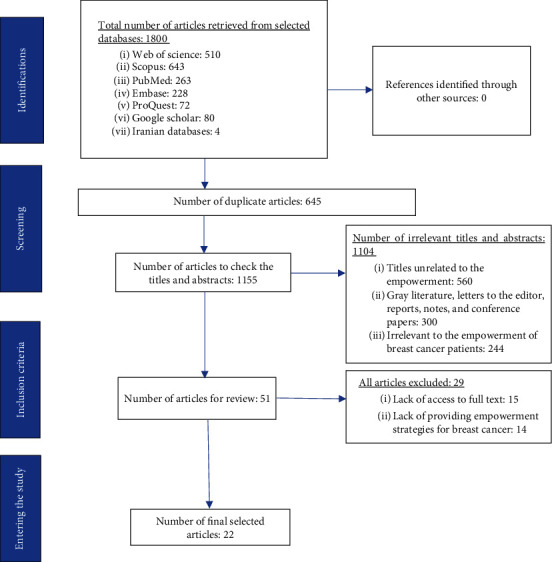
PRISMA flowchart of the included papers in the scoping review.

In the scoping review, 22 full-text articles were included in the present research (Figure 1). The bibliographic characteristics of those articles are presented in Table 5.

The studies had been conducted in Puerto Rico (*n* = 1), the Netherlands (*n* = 4), Finland (*n* = 1), Norway (*n* = 2), Iran (*n* = 5), South Korea (*n* = 1), the US (*n* = 6), Indonesia (*n* = 1), India (*n* = 2), and Nigeria (*n* = 1). The research methods of those studies included interventions (*n* = 14), interviews (*n* = 5), descriptive cross-sectional method (*n* = 2), and literature review (*n* = 1). Therefore, most of the studies had been conducted in the US, the Netherlands, and Iran and mainly with intervention and interview approaches. Accordingly, the main strategies extracted from the articles for empowering breast cancer women included the following: interactive empowerment and perceived social support; intrapersonal, individual, and behavioral psychological empowerment (mind-body-spirit connection balance through meditation, health visualization, and forgiveness; self-love, interaction through social media, and online groups and self-help groups; cancer survival and rehabilitation counseling, positive adjustment, education on coping, resilience, and post-traumatic stress; and positive view of the disease); empowerment through the formation of online support groups and local social networks (providing physiological and biological, functional, experimental, ethical, social, financial, and self-management information); cognitive-behavioral intervention through expert-guided self-help groups; family-centered empowerment; and spiritual empowerment (Table 5).

Table 6 shows the general results and strategies obtained from the scoping review as well as the interviews with the medical staff and the women with breast cancer.

## 4. Discussion

The present study is aimed at determining the empowerment status of breast cancer patients referred to the Shahid Motahari Breast Cancer Clinic in Iran, Shiraz, and at providing strategies to improve their empowerment in 2021 which was conducted in two phases.

The results of the quantitative phase showed that the majority of the studied patients were married, at a mean age of 48.7 years, with a mean disease duration of 3.7 years, having an average monthly income of 30.7 million rials (equal to 731 USD), not heads of households, with elementary school education, residents of Shiraz, unemployed, in stage 2 of the disease, without a history of any diseases, and living with their family. In Padmaja et al.'s study on the effectiveness of empowerment programs on breast cancer women, the patients had similar demographic characteristics, most of whom were 40-50 years of age and married, and had a personal job and elementary school education level [13].

The results of the present study showed that there was a significant relationship between monthly income and empowerment, and as the monthly income increased, the mean total score of the empowerment increased which can be due to that with the increase in their income, they had more financial support and could pay the direct and indirect costs of their disease personally. Furthermore, education level was significantly related to the mean total score of empowerment, so that the people with higher education levels were more capable than those with lower education levels. This could be due to having more knowledge, awareness, and information about the disease and self-care and trying to better understand the disease and its management through obtaining reliable information. These findings are confirmed by the results of the Stang and Mittelmark's study [41].

On the other hand, the mean and standard deviation of the empowerment items of the studied patients showed that most of them believed that their physicians were performing well and were reliable, and their families had played a supporting role for them. The majority of these patients were reluctant to use complementary therapies and followed their physicians' instructions. The mean dimensions of patient empowerment also showed that trust in the physician, family support, and spiritual beliefs could affect the empowerment of women with breast cancer, which is in line with the results of the studies by Lotfian et al. [46], Rostami et al. [47], Davarpanah et al. [48], and Elhani et al. [49].

Among the items of the empowerment scale, 11 items were not at the acceptable status. They included the patient's access to enough resources, the patient's access to required information, the relevance of the information available to the patient with the disease, the patient's willingness to be involved in decision-making related to the disease, the patient's ability to make decisions related to the disease, management of the disease outside the hospital by the physician, willingness of health professionals to involve the patient in making decisions on the disease, patient's ability to assist health professionals in making disease-related decisions, the use of complementary therapies, the importance of complementary therapies for patients to cope with the disease, and the feeling useful despite losing the job.

Among the items with a low mean in the first phase of the study was the patients' involvement in the treatment process, showing the importance of providing all treatment strategies to patients and making treatment decisions by them. One of the issues mentioned by the medical staff participating in the present study was the importance of beauty in women, which needed to be clearly explained to breast cancer patients before mastectomy. In this regard, Street and Voigt [50] in their study indicated that the patients who participated more actively in visits and consultations to make treatment decisions had greater control over such decisions than passive patients, and thus, they reported better health-related quality of life after treatment.

The patients also had a positive view of the physicians' willingness to involve the patient in clinical decision-making and their ability to manage the disease outside the hospital. This indicates the importance of effective communication between physicians and patients that needs to be considered in breast cancer treatment centers. The results of the scoping review and the interview phase also showed that empowerment strategies for breast cancer women were classified into five categories: financial support, informational support, interaction with the physician, occupational support, and complementary therapies. The most important financial support strategies for breast cancer women from the participants' point of view included bank loans, discounts on medical services, granting credit cards, continuing the treatment in the city of residence, health insurance coverage, donations, integrating all treatment procedures in the clinic, financial support of the government for cancer patients in their policies, proper distribution of medical centers in various cities, providing online services, and the use of different support groups.

It can be said that the costs of treating certain diseases such as breast cancer may impose a heavy financial burden on the patients and their families and relatives. In this regard, providing bank loans with long-term repayments for the patients could be a good strategy. Many treatments can also be performed in the city where the patient resides. In addition, increasing the insurance coverage for health services, especially chemotherapy drugs, can reduce many costs. Donor support for breast cancer patients and government policies for financial exemptions and lending, giving credit cards, attracting donor participation, and distributing medical services in different cities and centers can also be effective in financially empowering breast cancer women. Additionally, providing online services in the form of remote counseling, online appointments, etc., can reduce the travel costs of these patients to a large extent.

On the other hand, the patients' knowledge of breast cancer and its treatment methods could affect their capabilities. Providing reliable and accurate information is one of the necessities of empowering breast cancer women, which can be done through holding educational courses and providing training packages in the form of brochures and animations by the health centers, holding counseling sessions for patients' families, allocating sufficient time to the patients by physicians to provide them with sufficient information, teaching them the use of social networks and the Internet, making self-care groups in cyberspace or in person, teaching how to provide community-oriented services in the local language and culture, implementing educational programs based on the empowerment effective on breast cancer screening, holding educational sessions at the national as well as local and personal levels, teaching self-assessment skills, and patients' participation in self-help groups. Empowerment through the formation of online support groups in the local language was another strategy proposed by the participants to increase the patients' physiological and biological, functional, experimental, ethical, social, and financial knowledge, which could lead to increased self-management. In this regard, Lambrinou et al. carried out a study on the empowerment of diabetic patients and proposed providing self-management training to patients to manage disease conditions and improve their lifestyle [51], which is consistent with the results of the current study. Kondylakis et al. in their study addressed the need to create information and communication technology (ICT) infrastructures for empowering cancer patients and stated that these capabilities could be embedded in cancer patient empowerment platforms to increase cancer patients' resilience and coping abilities [52]. Similarly, another study showed that the creation of an information counseling system could help empower cancer patients. Such a system would provide the patient with the required information based on their conditions and would prevent confusion due to the large amount of information available on the Internet [53]. Accordingly, similar systems can be developed for breast cancer patients to access reliable, necessary, and sufficient health information [28, 35].

The results of the study by Moradi Manesh and Babakhani also showed that cognitive-behavioral empowerment had a positive impact on increasing breast cancer patients' self-efficacy and quality of life [54].

Furthermore, according to the results of the present study, breast self-assessment training and breast cancer screening can be effective in preventing the occurrence and progression of this disease, which is confirmed by the results of the study by Hassanpour and Alami [55]. Other similar studies showed the effective role of self-help groups in empowering breast cancer patients [6, 41].

One of the most important empowering strategies in terms of the informational support of breast cancer women was interactive empowerment and perceived social support. In this regard, the results of the study by Abadi Bavil and Dolatian showed that social support played a major role in adapting and coping with chronic and serious diseases such as cancer. In addition, social support was an influential factor in the mental health of the patients. The results also suggested that social support had a significant positive effect on the process of breast cancer treatment and recovery as it created an empathetic communication and a safety network for the patients and increased their adaptability to chronic diseases such as cancer. Social support also helped patients to feel better about themselves and be able to better deal with the disease. It also increased patients' self-confidence and hopeful thoughts, mental health, quality of life, and, consequently, the probability of their survival [56]. In a similar study, Firoozi et al. concluded that there was a significant relationship between self-empowerment skills and the quality of interpersonal interactions, and these variables could be good predictors of emotional disturbance in breast cancer patients [57].

The results of a study by Lee showed that holding disease management meetings, discussion groups, and telephone consultations for kidney patients had a favorable effect on their self-management, self-efficacy, and quality of life [58]. In a study, Sharf mentioned the role of online groups in information exchange, social support, decision-making, and preparation for breast cancer patients [45].

Zorrilla et al. in their study proposed an empowerment model for AIDS patients and stated that the model, which included six training sessions, could be used for breast cancer patients as well. The sessions included mindfulness training, forgiving oneself and others, providing updated information about the disease, lifestyle changes, and attending self-help groups [44], which is consistent with the results of the current study.

Numerous studies have also shown the role of family-based empowerment in reducing the adverse effects of diseases and increasing the patients' quality of life, some of which are the studies by Tozan et al. [59], Etemadifar et al. [60], Davarpanah et al. [48], and Hosseini et al. [30]. It is worth mentioning that the family-centered empowerment model has been emphasized more in other chronic diseases [61, 62] but could also be used in empowering breast cancer patients. The importance of this approach is that the illness of a family member affects the quality of life of others and it is necessary to provide required training to family members on how to treat and care for the sick member. In other words, providing information and education to the families of breast cancer women not only increases their health literacy in terms of the role of effective factors in the occurrence of the disease but also helps them treat the patient appropriately and understand her condition.

Similar to the present study, other researchers also emphasized the development of educational programs related to monthly regular and correct breast self-examinations and passing a training course to increase women's awareness of early detection of breast cancer [63, 64].

Spiritual empowerment of breast cancer patients was another proposed strategy; however, this approach has mainly been studied on the empowerment of the staff instead of the patients [46, 65].

According to the results of the present study, it was important for the patients to have a job and income to afford medical costs and have financial support, but employment conditions were not available for many of them. Thus, job creation for breast cancer patients willing to work could be a priority to help them not only meet their financial but also their spiritual needs and make them feel more useful. For cancer patients working during their illness, their organization managers should use strategies such as allowing them to use sick leave, supporting them in the form of delegating tasks commensurate with their physical and mental abilities, and providing a calm environment without work stress. They can also consider the teleworking strategy for the patients. Further studies are needed on the impact of employment on the life and treatment of chronic patients, including breast cancer ones.

According to the findings of this study, many patients did not have the experience of using complementary therapies such as psychiatric services, rehabilitation services, nutrition counseling, exercise, and traditional medicine despite their importance. Thus, it is suggested that the conditions for the correct use of these methods be provided under the supervision of their physicians in order to further empower the patients. In line with these results, van den Bergh et al. quoted Zimmerman and introduced psychological empowerment including interpersonal, behavioral, and interactive empowerment of cancer patients as one of the strategies for empowering them. They stated that empowering breast cancer patients could be mostly in the form of online patient education and through personal websites, peer groups, survivorship counseling, and professional self-help groups [39, 66].

In their study, van Uden-Kraan et al. pointed out the positive role of online support groups and concluded that such groups could help to exchange information, provide emotional support, gain experience, and entertain breast cancer patients. Through online support groups, patients could communicate better with physicians, the surrounding environment, and others, and their self-confidence, hope, and understanding of the disease, as well as the control over their lives and performance, would increase as well [43].

Like other studies, the present research had some limitations, which were the cross-sectional design of the study and the only use of questionnaires to determine the patients' empowerment status.

## 5. Conclusion

The results of the study showed that there were significant relationships between the mean empowerment score of the studied breast cancer patients and their income, education level, employment status, and having a job before the disease. It was also indicated that trust in the physician, family support, and spiritual beliefs could affect the empowerment of women with breast cancer. The results of this study classified the main strategies for empowering breast cancer women into five categories of financial support, informational support, interaction with the physician, occupational support, and complementary therapies, the use of which by the patients and their families, health policymakers, healthcare providers, and other stakeholders could help to improve the patients' quality of life while improving their empowerment.

Finally, future studies can be conducted to determine the appropriate models of financial empowerment and self-help groups for breast cancer patients; the effectiveness of providing remote medical services, consultation, and rehabilitation for these patients; the potential impact of the strategies mentioned on the patients' outcomes; and the potential challenges of implementing such strategies.

## Figures and Tables

**Table 1:** The means and standard deviations of age, monthly income, and illness duration of the subjects.

Variable	Mean ± SD	Minimum	Maximum
Age (year)	48.7 ± 10.26	27	76
Monthly income (million rials)	30.72 ± 1.93	2	11
Illness duration (year)	3.63 ± 3.52	One month	20 years

**Table 2:** Demographic and clinical characteristics of the studied patients in the quantitative phase.

Variables		Number of patients	%
Marital status	Single	56	18.1
	Married	254	81.9
	Total	310	100

How to live	Alone	11	3.5
	With family	299	96.5
	Total	310	100

Being head of household	Yes	37	11.9
	No	273	88.1
	Total	310	100

Education level	Illiterate	23	7.4
	Elementary school	234	75.5
	Academic	53	17.1
	Total	310	100

Residence	Local	142	45.8
	Nonlocal	168	54.2
	Total	310	100

Employment status	Employed	43	3.5
	Retired	11	13.9
	Unemployed	256	85.5
	Total	310	100

Stage of the disease	Stage 1	14	4.5
	Stage 2	23	7.4
	Stage 3	7	2.3
	Missing	266	85.8
	Total	310	100

History of other diseases	Yes	68	21.9
	No	242	78.1
	Total	310	100

Having a job before the disease	Yes	62	19.9
	No	249	80.1
	Total	310	100

**Table 3:** Mean scores of dimensions of empowerment of the studied breast cancer patients.

	Dimensions	Mean	SD
1	Recourses	3.42	0.82
2	Information	3.33	0.95
3	Participation in decision-making	3.25	1.09
4	Family support	4.28	0.57
5	Support of friends	3.78	0.99
6	Interaction with the physician	4.36	0.53
7	Patient perception of the physician's ability to manage the disease	3.20	0.57
8	Patient perception of health professionals' willingness to let him/her participate in decision-making	2.53	1.03
9	Complementary therapies	1.93	0.88
10	Spiritual beliefs	4.19	0.88
11	Acceptance and adaptation to disease	4.15	0.66
12	Patient perception of usefulness to friends	3.99	0.83
13	Patient perception of usefulness to family	4.13	0.67
14	Having a job	3.52	1.30
	Total	3.58	0.41

**Table 4:** The unacceptable status of empowerment items for the studied breast cancer patients.

Items	Mean	Status
I have enough resources to manage my disease.	2.95	Unacceptable
I have access to all the information needed to manage my disease.	3.30	Unacceptable
The information I have is related to my disease.	3.31	Unacceptable
I want to be involved in decisions related to my disease.	3.18	Unacceptable
I am able to make decisions om my disease.	3.27	Unacceptable
My physician is able to manage my disease outside the hospital (my physician can follow up my disease outside the hospital).	1.99	Unacceptable
Health professionals welcome my involvement in making decisions on my disease.	2.29	Unacceptable
I am able to assist health professionals in making decisions on my disease.	2.70	Unacceptable
I use complementary therapies (such as traditional medicine or rehabilitation).	1.87	Unacceptable
Complementary therapies help me cope with my disease (complementary therapies such as traditional medicine or rehabilitation).	1.95	Unacceptable
Although I have lost my job, I still feel useful.	2.65	Unacceptable

**Table 5:** Descriptive characteristics of the selected studies.

The first author (year)	Country	Title	Research method	Main result and empowerment strategy
Padmaja et al. [13]	India	Effectiveness of empowerment program on breast cancer and education on breast self-examination among women in selected rural areas/ST, AP Tirupati, India	Intervention	Training and improving breast self-assessment skills can be effective in the early diagnosis and early treatment of cancer.
Mustika et al. [27]	Indonesia	Community empowerment through the cervical and breast cancer early detection program with the Formation of Srikandi Cadres (Early Cancer Awareness) in Kangean Islands, Sumenep Regency	Intervention	Training to provide community-based services in the local language and culture is useful for increasing women's knowledge of breast self-assessment and performing acetic acid imaging to diagnose and reduce breast and cervical cancer.
Molina et al. [28]	The US	The “Empowering Latinas to obtain breast cancer screenings” study: rationale and design	Intervention	Empowering and educating women through social media can help familiarize them with the importance of breast cancer screening.
Hanson et al. [29]	Nigeria	Knowledge and practice of breast self-examination among rural women in southwestern Nigeria: Implications for the development of a women's empowerment program	Interview	Breast self-assessment educational intervention can help with early diagnosis and treatment of the disease.
Shin and Park [17]	South Korea	Effect of empowerment on the quality of life of the survivors of breast cancer: The moderating effect of self-help group participation	Descriptive cross-sectional	Participation in self-help groups has an important impact on breast cancer survivors' sense of empowerment and, consequently, their quality of life.
Hosseini et al. [30]	Iran	The impact of the implementation of the empowerment family-centered model on the symptom scales of the lives quality of women with breast cancer undergoing chemotherapy	Intervention	Family-based empowerment of women with breast cancer reduces the adverse effects of chemotherapy and improves their quality of life.
Lee et al. [31]	The US	Direct interactive public education by breast radiologists about screening mammography: impact on anxiety and empowerment	Intervention	Holding training sessions on the importance of mammography screening can reduce anxiety and cause informed decision-making in breast cancer patients.
Pashaee et al. [32]	Iran	The impact of the implementation of the empowerment family-centered model on the performance scale of the lives quality of women with breast cancer undergoing chemotherapy	Intervention	Family-based empowerment in women with breast cancer is effective in reducing the adverse effects of treatment and improving their quality of life.
van den Berg et al. [33]	The Netherlands	BREATH: web-based self-management for psychological adjustment after primary breast cancer--results of a multicenter randomized controlled trial	Intervention	Internet-based self-management can reduce the psychological pressure of breast cancer.
Sadati et al. [34]	Iran	Religion as an empowerment context in the narrative of women with breast cancer	Interview	Spiritual empowerment includes strengthening one's spiritual soul and faith, which leads to the resumption of the person's normal life and not considering breast cancer as the end of life.
Kaur and Bisht [35]	India	Assessing the impact of awareness program on breast and cervical cancer knowledge empowerment among working women in education sector	Interview	Holding educational sessions at national, local, and individual levels can increase patients' awareness of diagnostic and prevention methods of breast cancer.
Gabitova and Burke [36]	The US	Improving healthcare empowerment through breast cancer patient navigation: a mixed methods evaluation in a safety-net setting	Intervention	Patient navigation in the breast clinic had a positive impact on patients' experiences with care and healthcare empowerment.
Ryhänen et al. [37]	Finland	The impact of an empowering Internet-based Breast Cancer Patient Pathway program on breast cancer patients' clinical outcomes: a randomised controlled trial.	Intervention	Internet-based patient participation helps to empower the patient in six dimensions: physiological and biological, functional, experimental, ethical, social, and financial.
Martinez-Ramos et al. [38]	The US	Quality of life of Latina breast cancer survivors: from silence to empowerment	Interview	Family support and peer advocacy helped survivors help to reduce the fear of cancer recurrence, fight the fear of social stigma, become stronger in the face of ongoing life changes, and strive to provide healthcare and social support for breast cancer survivors.
van den Berg et al. [39]	The Netherlands	The cancer empowerment questionnaire: Psychological empowerment in breast cancer survivors	Descriptive cross-sectional	Psychological empowerment includes the patient interpersonal, behavioral, and interactive empowerment. Empowerment of breast cancer patients is mostly in the form of online patient education, personal websites, peer groups, survival counseling, cancer rehabilitation, professional self-help groups, social cognitive processing and interactive empowerment, positive adjustment, coping, resilience, optimism, personal control, post-traumatic growth, and perceived social support.
van den Berg et al. [40]	India	Rationale of the breast cancer e-health [BREATH] multicentre randomised controlled trial: an internet-based self-management intervention to foster adjustment after curative breast cancer by decreasing distress and increasing empowerment	Intervention	The use of BREATH Internet-based intervention leads to psychological self-management in breast cancer survivors. In this method, the patient's own capability is used. The cost-effectiveness of this method is high, and it is very affordable. This method provides emotional, physical, and social rehabilitation to breast cancer survivors. In addition, cognitive behavioral therapy, self-management, printed educational materials, and educational videos prepared by peers are effective in empowering breast cancer patients.
Stang and Mittelmark [41]	Norway	Intervention to enhance empowerment in breast cancer self-help groups	Intervention	Self-help groups led by peers or specialists can affect the rehabilitation of breast cancer women. Psychological empowerment includes intrapersonal, interpersonal, and behavioral empowerment.
Fisher and Howell [42]	The US	The power of empowerment: An ICF-based model to improve self-efficacy and upper extremity function of survivors of breast cancer. Rehabilitation Oncology	Review	A combination of empowerment and self-efficacy of breast cancer survivors affects the personal factors based on the International Classification of Functioning (ICF) model.
Stang and Mittelmark [6]	Norway	Learning as an empowerment process in breast cancer self-help groups	Intervention	The learning process in expert-led self-help groups increases awareness, gaining objective knowledge, learning from the experiences of others, and acquiring new perspectives on oneself and life.
van Uden-Kraan et al. [43]	The Netherlands	Empowering processes and outcomes of participation in online support groups for patients with breast cancer, arthritis, or fibromyalgia	Interview	Participation of breast cancer patients in online support groups empowers them through increased self-confidence, trust in the physician and treatment, social environment, disease acceptance, optimism and self-control, participation in community activities, and a sense of social well-being.
Zorrilla et al. [44]	Puerto Rico	An empowerment intervention for women living with HIV and its adaptation for women with a diagnosis of breast cancer	Intervention	The biopsychological model includes patient participation in six full-day workshops in which multiple biopsychosocial dimensions are explored within the group, and diverse experiential activities are carried out related to the day's topics.
Sharf [45]	The US	Communicating breast cancer on-line: support and empowerment on the Internet	Intervention	Online groups are involved in increasing the empowerment of women with breast cancer. By exchanging information in these groups, people can make good decisions on their treatment process.

**Table 6:** Strategies to meet the empowerment needs of breast cancer women.

Theme	Strategies to meet the empowerment needs of breast cancer women				
Subtheme	Financial support	Informational support	Interaction with the physician	Occupational support	Complementary therapies
Final code	Giving bank loans Applying some medical discounts Granting credit cards Continuing treatment in the city of residence Health insurance coverage Donations Integrating all procedures in the clinic Government's financial support for cancer patients in their policies Providing online appointment facilities and remote visits Proper distribution of medical centers in cities Using social and family support groups	Producing educational content in the form of animations Making self-help groups to exchange information Using educational pamphlets and brochures Teaching patients the skills of using the Internet Informing those around them Establishment of training units in medical centers Using physicians as the main source of information	Online visits Use of support team Direct communication with patients The patient affairs follow-up program Increasing the quality of interpersonal relationships and self-efficacy Surveying breast cancer patients	Sick leave Support of senior executives of the organization for cancer patients Giving fewer responsibilities to working patients Teleworking	Using a psychiatrist Providing rehabilitation services to patients Using nutrition counseling Using group therapy Using exercise as a complementary treatment Using traditional medicine as a complementary treatment Using psychological empowerment Strengthening the individual's spiritual spirit and faith Using a biological-psychological model

	Using Internet-based intervention (BREATH)	Holding educational courses for patients Participation in online groups Teaching how to provide community-oriented services in the local language and culture Implementing empowerment-based educational programs affecting breast cancer screening Education through social networks Self-help groups Holding educational sessions on the importance of screening Holding educational sessions at national, local, and individual levels Teaching self-assessment skills			

## Data Availability

The datasets used and/or analyzed during the current study are available from the corresponding author on reasonable request.

## Supplementary Materials

Supplementary materials Table S1: the search strategy used in the present study. Table S2: the search strings used in the search strategy.
