# Supplementary material for: Strategies to Promote Empowerment Status of Breast Cancer Women
Source: Int J Breast Cancer. 2024 Feb 5;2024:3305399. doi: 10.1155/2024/3305399 (PMC10861285; doi:10.1155/2024/3305399)
Supplement: Supplementary materials — Table S1: the search strategy used in the present study. Table S2: the search strings used in the search strategy. [file 3305399.f1.docx]

| **Table S1: The search Strategy used in the present study** | |
| --- | --- |
| **Search Engines and Databases**: Magiran, Irandoc, SID, Web of Science, Scopus, Pubmed, Embase, ProQuest, Google Scholar | |
| **Limits**: Language (resources with at least an abstract in English and Persian) | |
| **Date:** up to 31 April 2021 | |
| **Strategy:** #1 AND #2 | |
| **OR** Malignant Neoplasm of Breast **OR** Breast Malignant Neoplasm OR Breast Malignant Neoplasms **OR** Malignant Tumor of Breast **OR** Breast Malignant Tumor **OR** Cancer of Breast **OR** Mammary Neoplasm **OR** Human Mammary Neoplasm **OR** Mammary Neoplasm | #1 |
| Empowerment **OR** power **OR** psychological empowerment **OR** cancer empowerment **OR** healthcare empowerment **OR** patient empowerment **OR** psychological outcome | #2 |

| **Table S2: The search strings used in the search strategy** | | |
| --- | --- | --- |
| **Databases** | **Sample search strategy** | **Results** |
| PubMed | (“Breast Neoplasm”[tiab] OR (Neoplasm[tiab] AND Breast[tiab]) OR “Breast Tumors”[tiab] OR “Breast Tumor”[tiab] OR (Tumor[tiab] AND Breast[tiab]) OR (Tumors[tiab] AND Breast[tiab]) OR (Neoplasms[tiab] AND Breast[tiab]) OR “Breast Cancer”[tiab] OR (Cancer[tiab] AND Breast[tiab]) OR “Mammary Cancer”[tiab] OR (Cancer[tiab] AND Mammary[tiab]) OR (Cancers[tiab] AND Mammary[tiab]) OR “Mammary Cancers”[tiab] OR “Malignant Neoplasm of Breast”[tiab] OR “Breast Malignant Neoplasm”[tiab] OR “Breast Malignant Neoplasms”[tiab] OR “Malignant Tumor of Breast”[tiab] OR “Breast Malignant Tumor”[tiab] OR “Breast Malignant Tumors”[tiab] OR “Cancer of Breast”[tiab] OR “Cancer of the Breast”[tiab] OR (“Mammary Carcinoma”[tiab] AND Human[tiab]) OR (Carcinoma[tiab] AND “Human Mammary”[tiab]) OR (Carcinomas[tiab] AND “Human Mammary”[tiab]) OR “Human Mammary Carcinomas”[tiab] OR (“Mammary Carcinomas”[tiab] AND Human[tiab]) OR “Human Mammary Carcinoma”[tiab] OR (“Mammary Neoplasms”[tiab] AND Human[tiab]) OR “Human Mammary Neoplasm”[tiab] OR “Human Mammary Neoplasms”[tiab] OR (Neoplasm[tiab] AND “Human Mammary”[tiab]) OR (Neoplasms[tiab] AND “Human Mammary”[tiab]) OR (“Mammary Neoplasm”[tiab] AND Human[tiab]) OR “Breast Carcinoma”[tiab] OR “Breast Carcinomas”[tiab] OR (Carcinoma[tiab] AND Breast[tiab]) OR (Carcinomas[tiab] AND Breast[tiab])) AND (empowerment[tw] OR (power[tw] AND psychological[tw]) OR “psychological empowerment”[tw] OR “cancer empowerment”[tw] OR “healthcare empowerment”[tw] OR “patient empowerment”[tw] OR “psychological outcome”[tw]) | 263 |
| Embase | (“Breast Neoplasm”:ti,ab OR (Neoplasm:ti,ab AND Breast:ti,ab) OR “Breast Tumors”:ti,ab OR “Breast Tumor”:ti,ab OR (Tumor:ti,ab AND Breast:ti,ab) OR (Tumors:ti,ab AND Breast:ti,ab) OR (Neoplasms:ti,ab AND Breast:ti,ab) OR “Breast Cancer”:ti,ab OR (Cancer:ti,ab AND Breast:ti,ab) OR “mammary cancer”:ti,ab OR (Cancer:ti,ab AND Mammary:ti,ab) OR (Cancers:ti,ab AND Mammary:ti,ab) OR “Mammary Cancers”:ti,ab OR “Malignant Neoplasm of Breast”:ti,ab OR “Breast Malignant Neoplasm”:ti,ab OR “Breast Malignant Neoplasms”:ti,ab OR “Malignant Tumor of Breast”:ti,ab OR “Breast Malignant Tumor”:ti,ab OR “Breast Malignant Tumors”:ti,ab OR “Cancer of Breast”:ti,ab OR “Cancer of the Breast”:ti,ab OR (“Mammary Carcinoma”:ti,ab AND Human:ti,ab) OR (Carcinoma:ti,ab AND “Human Mammary”:ti,ab) OR (Carcinomas:ti,ab AND “Human Mammary”:ti,ab) OR “Human Mammary Carcinomas”:ti,ab OR (“Mammary Carcinomas”:ti,ab AND Human:ti,ab) OR “Human Mammary Carcinoma”:ti,ab OR (“Mammary Neoplasms”:ti,ab AND Human:ti,ab) OR “Human Mammary Neoplasm”:ti,ab OR “Human Mammary Neoplasms”:ti,ab OR (Neoplasm:ti,ab AND “Human Mammary”:ti,ab) OR (Neoplasms:ti,ab AND “Human Mammary”:ti,ab) OR (“Mammary Neoplasm”:ti,ab AND Human:ti,ab) OR “Breast Carcinoma”:ti,ab OR “Breast Carcinomas”:ti,ab OR (Carcinoma:ti,ab AND Breast:ti,ab) OR (Carcinomas:ti,ab AND Breast:ti,ab)) AND (empowerment/exp OR (power/exp AND psychological/exp) OR “psychological empowerment”/exp OR “cancer empowerment”/exp OR “healthcare empowerment”/exp OR “patient empowerment”/exp OR “psychological outcome”/exp) | 228 |
| Scopus | (TITLE-ABS-KEY(“Breast Neoplasm”) OR (TITLE-ABS-KEY(Neoplasm) AND TITLE-ABS-KEY(Breast)) OR TITLE-ABS-KEY(“Breast Tumors”) OR TITLE-ABS-KEY(“Breast Tumor”) OR (TITLE-ABS-KEY(Tumor) AND TITLE-ABS-KEY(Breast)) OR (TITLE-ABS-KEY(Tumors) AND TITLE-ABS-KEY(Breast)) OR (TITLE-ABS-KEY(Neoplasms) AND TITLE-ABS-KEY(Breast)) OR TITLE-ABS-KEY(“Breast Cancer”) OR (TITLE-ABS-KEY(Cancer) AND TITLE-ABS-KEY(Breast)) OR TITLE-ABS-KEY(“mammary cancer”) OR (TITLE-ABS-KEY(Cancer) AND TITLE-ABS-KEY(Mammary)) OR (TITLE-ABS-KEY(Cancers) AND TITLE-ABS-KEY(Mammary)) OR TITLE-ABS-KEY(“Mammary Cancers”) OR TITLE-ABS-KEY(“Malignant Neoplasm of Breast”) OR TITLE-ABS-KEY(“Breast Malignant Neoplasm”) OR TITLE-ABS-KEY(“Breast Malignant Neoplasms”) OR TITLE-ABS-KEY(“Malignant Tumor of Breast”) OR TITLE-ABS-KEY(“Breast Malignant Tumor”) OR TITLE-ABS-KEY(“Breast Malignant Tumors”) OR TITLE-ABS-KEY(“Cancer of Breast”) OR TITLE-ABS-KEY(“Cancer of the Breast”) OR (TITLE-ABS-KEY(“Mammary Carcinoma”) AND TITLE-ABS-KEY(Human)) OR (TITLE-ABS-KEY(Carcinoma) AND TITLE-ABS-KEY(“Human Mammary”)) OR (TITLE-ABS-KEY(Carcinomas) AND TITLE-ABS-KEY(“Human Mammary”)) OR TITLE-ABS-KEY(“Human Mammary Carcinomas”) OR (TITLE-ABS-KEY(“Mammary Carcinomas”) AND TITLE-ABS-KEY(Human)) OR TITLE-ABS-KEY(“Human Mammary Carcinoma”) OR (TITLE-ABS-KEY(“Mammary Neoplasms”) AND TITLE-ABS-KEY(Human)) OR TITLE-ABS-KEY(“Human Mammary Neoplasm”) OR TITLE-ABS-KEY(“Human Mammary Neoplasms”) OR (TITLE-ABS-KEY(Neoplasm) AND TITLE-ABS-KEY(“Human Mammary”)) OR (TITLE-ABS-KEY(Neoplasms) AND TITLE-ABS-KEY(“Human Mammary”)) OR (TITLE-ABS-KEY(“Mammary Neoplasm”) AND TITLE-ABS-KEY(Human)) OR TITLE-ABS-KEY(“Breast Carcinoma”) OR TITLE-ABS-KEY(“Breast Carcinomas”) OR (TITLE-ABS-KEY(Carcinoma) AND TITLE-ABS-KEY(Breast)) OR (TITLE-ABS-KEY(Carcinomas) AND TITLE-ABS-KEY(Breast))) AND (TITLE-ABS-KEY(empowerment) OR (TITLE-ABS-KEY(power) AND TITLE-ABS-KEY(psychological)) OR TITLE-ABS-KEY(“psychological empowerment”) OR TITLE-ABS-KEY(“cancer empowerment”) OR TITLE-ABS-KEY(“healthcare empowerment”) OR TITLE-ABS-KEY(“patient empowerment”) OR TITLE-ABS-KEY(“psychological outcome”)) | 643 |
| Web of Science | (TS=(“Breast Neoplasm”) OR (TS=(Neoplasm) AND TS=(Breast)) OR TS=(“Breast Tumors”) OR TS=(“Breast Tumor”) OR (TS=(Tumor) AND TS=(Breast)) OR (TS=(Tumors) AND TS=(Breast)) OR (TS=(Neoplasms) AND TS=(Breast)) OR TS=(“Breast Cancer”) OR (TS=(Cancer) AND TS=(Breast)) OR TS=(“mammary cancer”) OR (TS=(Cancer) AND TS=(Mammary)) OR (TS=(Cancers) AND TS=(Mammary)) OR TS=(“Mammary Cancers”) OR TS=(“Malignant Neoplasm of Breast”) OR TS=(“Breast Malignant Neoplasm”) OR TS=(“Breast Malignant Neoplasms”) OR TS=(“Malignant Tumor of Breast”) OR TS=(“Breast Malignant Tumor”) OR TS=(“Breast Malignant Tumors”) OR TS=(“Cancer of Breast”) OR TS=(“Cancer of the Breast”) OR (TS=(“Mammary Carcinoma”) AND TS=(Human)) OR (TS=(Carcinoma) AND TS=(“Human Mammary”)) OR (TS=(Carcinomas) AND TS=(“Human Mammary”)) OR TS=(“Human Mammary Carcinomas”) OR (TS=(“Mammary Carcinomas”) AND TS=(Human)) OR TS=(“Human Mammary Carcinoma”) OR (TS=(“Mammary Neoplasms”) AND TS=(Human)) OR TS=(“Human Mammary Neoplasm”) OR TS=(“Human Mammary Neoplasms”) OR (TS=(Neoplasm) AND TS=(“Human Mammary”)) OR (TS=(Neoplasms) AND TS=(“Human Mammary”)) OR (TS=(“Mammary Neoplasm”) AND TS=(Human)) OR TS=(“Breast Carcinoma”) OR TS=(“Breast Carcinomas”) OR (TS=(Carcinoma) AND TS=(Breast)) OR (TS=(Carcinomas) AND TS=(Breast))) AND (TS=(empowerment) OR (TS=(power) AND TS=(psychological)) OR TS=(“psychological empowerment”) OR TS=(“cancer empowerment”) OR TS=(“healthcare empowerment”) OR TS=(“patient empowerment”) OR TS=(“psychological outcome”)) | 510 |
| ProQuest | TI,AB,SU(“Breast Neoplasm” OR (Neoplasm AND Breast) OR “Breast Tumors” OR “Breast Tumor” OR (Tumor AND Breast) OR (Tumors AND Breast) OR (Neoplasms AND Breast) OR “Breast Cancer” OR (Cancer AND Breast) OR “mammary cancer” OR (Cancer AND Mammar) OR (Cancers AND Mammary) OR “Mammary Cancers” OR “Malignant Neoplasm of Breast” OR “Breast Malignant Neoplasm” OR “Breast Malignant Neoplasms” OR “Malignant Tumor of Breast” OR “Breast Malignant Tumor” OR “Breast Malignant Tumors” OR “Cancer of Breast” OR “Cancer of the Breast” OR (“Mammary Carcinoma” AND Human) OR (Carcinoma AND “Human Mammary”) OR (Carcinomas AND “Human Mammary”) OR “Human Mammary Carcinomas” OR (“Mammary Carcinomas” AND Huma) OR “Human Mammary Carcinoma” OR (“Mammary Neoplasms” AND Human) OR “Human Mammary Neoplasm” OR “Human Mammary Neoplasms” OR (Neoplasm AND “Human Mammary”) OR (Neoplasms AND “Human Mammary”) OR (“Mammary Neoplasm” AND Human) OR “Breast Carcinoma” OR “Breast Carcinomas” OR (Carcinoma AND Breast) OR (Carcinomas AND Breast)) AND TI,AB,SU(empowerment OR (power AND psychological) OR “psychological empowerment” OR “cancer empowerment” OR “healthcare empowerment” OR “patient empowerment” OR “psychological outcome”) | 72 |
| Google Scholar | Titles: breast cancer, empowerment | 80 |
| Iranian databases (SID, Magiran, Irandoc) | Titles: Empowerment, breast cancer | 4 |
